# Variability in Post-Discharge Mortality Rates and Predictors over Time: Data from a Five-Year Ward-Wide Study

**DOI:** 10.3390/jcm15020850

**Published:** 2026-01-20

**Authors:** Giuseppe A. Ramirez, Bruno N. Germinario, Giovanni Benanti, Pier Francesco Caruso, Francesca Mette, Gaia Pagliula, Adriana Cariddi, Silvia Sartorelli, Elisabetta Falbo, Alessandro Marinosci, Francesca Farina, Giacomo Pacioni, Elena Rela, Pietro Barbieri, Moreno Tresoldi, Enrica P. Bozzolo

**Affiliations:** 1 Unit of Immunology, Rheumatology, Allergy and Rare Diseases, IRCCS Ospedale San Raffaele, via Olgettina 60, 20132 Milan, Italy; 2 Faculty of Medicine, Università Vita-Salute San Raffaele, via Olgettina 58, 20123 Milan, Italy; 3 Unit of General Medicine and Advanced Care, IRCCS Ospedale San Raffaele, via Olgettina 60, 20132 Milan, Italymarinosci.alessandro@hsr.it (A.M.); bozzolo.enrica@hsr.it (E.P.B.); 4 Clinical Management Unit, IRCCS Ospedale San Raffaele, via Olgettina 60, 20132 Milan, Italy

**Keywords:** after discharge, complexity, frailty, mortality rates, palliative care

## Abstract

**Background/Objectives:** Patients with complex chronic disorders constitute a growing share of the general population and are frequently hospitalised for acute care in Internal Medicine Departments. Little is known about long-term rates and predictors of post-discharge mortality, possibly contributing to suboptimal and discontinuous care, including delayed referral to palliative programmes. **Methods:** To assess the long-term post-discharge mortality of patients admitted to Internal Medicine Departments and its predictors, we analysed a cohort of old, multi-morbid subjects, corresponding to the whole population of patients admitted to an Internal Medicine Department over 12 months (February 2016–March 2017). Public health registries were interrogated to assess the five-year mortality (up to 2022) of patients discharged alive. **Results:** Post-discharge mortality was 57% at follow-up end, with an early peak rate of 32% at year 1, a 10–14% intermediate rate at years 2–4, and a 7% late rate, approaching expected figures in the general population. Cancer, neurological and liver disorders, and respiratory failure were significantly associated with early and intermediate mortality, while renal disorders, dependence for daily activities, and immunodepression were selectively relevant for death in the first year. Cardiovascular and upper gastrointestinal disorders were associated with late mortality. Surrogate measures of frailty, intensity of care, and patient complexity were also able to predict early-, intermediate-, and late-mortality risk. **Conclusions:** A relevant fraction of patients hospitalised in Internal Medicine Departments might require palliative care. Dissecting the differential contribution of clinical and healthcare-associated variables for short, medium-, and long-term mortality might facilitate patient management and identify subjects in need of early or simultaneous palliative care.

## 1. Introduction

Population aging, social fragmentation, and inadequate adaptation of healthcare systems to changing demographic scenarios have generated a novel, heterogeneous group of patients, characterised by multimorbidity, exposure to multiple drugs and insufficient connection with the healthcare/social network [1,2,3,4,5,6]. Public health countermeasures to quantitatively higher and qualitatively more complex needs are further exacerbated by unfavourable demographics. In fact, countries with higher rates of population aging, such as Italy, show dependency indexes (that is, ratios of non-working-age individuals/working population) that are already beyond the population capacity to self-sustain by generating sufficient wealth and workforce to respond to workload demands [7,8]. Regressive policies supposedly aimed at prioritising individual/local wealth protection and non-welfare investments at the expense of public health service cohesion and support constitute additional maladaptive responses to the critical problems posed by population aging and by the surge in prevalence of chronic complex frail (CCF) patients [9,10,11,12,13,14]. CCF patients often do not fit any classification based on classical medical specialties and are therefore generically referred to Internal Medicine Departments for in- and outpatient care [1,15]. In this context, contrasting acute decompensation of chronic diseases is mistakenly intended as the main objective of therapeutic intervention, and acute services are used as surrogate solutions for chronic needs. This phenomenon has measurable limitations. Hospitalisation and intensive care increase the risk of further morbidity by falls, nosocomial infections, and other complications [16,17], which may prolong in-hospital stay and eventually increase mortality among frail patients [5,18]. Complicated hospitalisations, in turn, are associated with higher economic costs, further jeopardising healthcare system sustainability [19,20].

While cancer-related frailty is well known and cancer-specific paths of care are better delineated, management strategies for non-cancer CCF patients are less defined, possibly resulting in suboptimal care [21,22,23]. Limited knowledge about non-cancer CCF patient survival trajectories inside and outside hospital settings in “natural history” might account for restricted patient access to appropriate healthcare services, including early palliative care and/or simultaneous palliative care programmes [24,25]. While palliative care is generally defined as a comprehensive type of medical care aiming at maximising quality of life and minimising suffering for patients with life-threatening conditions and/or limited predicted lifespan [26], early palliative care refers to practices aiming at accelerating the recognition of patients in need of palliative care along with their access to palliative care programmes, and simultaneous palliative care is the practice of providing therapies selectively aiming at psychological and physical relief alongside curative treatments [27]. These strategies may not only prove effective for quality of life, symptom control, patient and caregiver satisfaction, and costs but also for overall survival [27,28,29]. Previous studies have shown that, besides age and cancer, measures of illness severity across multiple functional domains are major predictors of hospital mortality in acute care along with factors related to healthcare network discontinuity or stress such as admittance from nursing homes, admittance to peripheral hospitals, or reduced personnel [20,30,31]. Few studies have investigated patient trajectories after hospital discharge, with most of them focusing on relatively short observation timeframes and small samples of selected patients while assuming that the impact of distinct clinical variables would remain constant at different stages of patient journeys after hospitalisation [32,33].

Aiming at contributing to filling this knowledge gap, we performed a five-year post-hospitalisation follow-up study on a cohort of patients admitted to an Internal Medicine Department and investigated early and late mortality rates and their predictors over time.

## 2. Materials and Methods

### 2.1. Original Study Cohort

We leveraged on a 12-month-long observational prospective study (“SIM”) investigating the clinical phenotype and in-hospital outcomes of all patients admitted to a University Hospital Internal Medicine Department [20] and analysed their survival in the following five years with the aim of measuring long-term post-discharge trajectories and defining potential descriptors/determinants of residual mortality risk beyond the first months after hospitalisation. During hospitalisation, clinical and healthcare-related variables were collected from the whole population of patients admitted to the Unit in 2016–2017. Based on this, we derived individual and unit-average values during (a) single days, (b) the whole observation timeframe, (c) individual patient hospitalisation timeframes. Average values were rounded to the nearest integer. Consequently, each individual hospitalisation trajectory encompassed a first set of data referring to each patient’s characteristics and another one summarising the features of the whole Unit environment at the time of each patient’s hospitalisation course (Supplementary Figure S1). The only inclusion criterion was being admitted into the Unit within the study timeframe. There were no exclusion criteria. All subjects were enrolled with informed consent under the SIM (“Sostenibilità In Medicina”) protocol, approved by San Raffaele Ethics Committee, Milan, Italy (ref. 110/INT/2015) on 8 October 2015 and conforming to the Declaration of Helsinki.

#### 2.1.1. Clinical Variables

Patient data collected during hospitalisation included demographics (sex, age), variations in prevalence and severity of multiple causes of morbidity, clinical features at presentation and during hospitalisation, length of stay, and overall survival. We referred to the definitions of the Cumulative Illness Rating Scale (CIRS) [34] parameters to dichotomously record cardiac, lung, kidney, liver, endocrine–metabolic, vascular/haematological, upper and lower gastrointestinal, genitourinary, ear–nose–throat–eye, musculoskeletal, skin, psychiatric, and neurological disorders in the patients’ history. Each CIRS component was also scored from 0 to 4, and cumulative scores were used to derive the total CIRS score. The total CIRS score and its severity (CIRS-SI) and comorbidity (CIRS-CI) sub-indices were used to measure patient complexity. We also recorded whether patients had a history of cancer and graded it on an arbitrary 0–4 scale based on their predicted prognosis: patients with an expected prognosis of less than six months were assigned the highest (4) score; patients with six months to five years, 5–10 years, and >10 years expected prognosis were assigned 3, 2, 1, and 0 points, respectively. Additional dichotomous variables included a history of immune-mediated disorders, inborne or acquired causes of immunodepression, and presentation or late occurrence of infections during hospitalisation. Nosocomial infections were distinguished from infections at presentation by considering all events presenting at least 48 h after admission as hospital-acquired infections. Dependence on activity of daily living (ADL)/instrumental ADL (IADL) was defined as an inability to autonomously walk, care for personal hygiene, maintain continence, feed, move from bed, and/or manage daily oral medications. To obtain a simplified objective measurement of patient frailty, we constructed a 20-item frailty index (FI) as per [35] (Supplementary Table S1). Patients with FI > 0.25 were considered frail [36].

#### 2.1.2. Assistance-Related Variables

Intensity of care was quantitated with the Nine Equivalents of nursing Manpower Score (NEMS) [37]. This score records the need for continuous measuring of vital signs (including fluid balance), oxygen supplementation with or without non-invasive mechanical ventilation, renal replacement therapy, intravenous treatments, treatment with one or more vasoactive drugs, non-routine procedures performed in the reference ward and exits from the ward for additional diagnostics or treatments. NEMS components were also analysed separately and used to calculate individual and unit-average numbers of non-routine procedures performed in the Unit and of exits from the ward for diagnostic or treatment procedures (Supplementary Figure S1). Assistance-related variables also included the number of senior physicians in service for each analysed day.

### 2.2. Public Health Data

We accessed the demographic registry of the Region of Lombardy, Italy, which provides updated patient-level information about the survival status of Lombardy residents. We retrieved data up to five years after discharge. Extended data for patients surviving after five years was also available up to August 2022 (extended follow-up). Data regarding non-Lombardy residents were unavailable. To compare observed mortality rates with the expected rates in the general population, we also retrieved data from the Italian National Institute of Statistics (ISTAT) database (www.istat.it, accessed on 16 February 2025), where mortality probabilities are calculated annually for each year of age based on the real number of deaths. We retrieved ISTAT data from 2017 to 2022 and calculated annual average expected mortality rates for a population with the same age distribution as in our cohort. Specifically, each annual average was weighted on the number of subjects with a given age during the year of interest.

### 2.3. Outcomes and Timeframes of Interest

We analysed post-hospitalisation deaths occurring during the five years following hospital discharge and calculated mortality rates: (a) per year; (b) across the whole timeframe of observation. Based on mortality frequency during follow-up, we also subdivided the five-year window into an early (year 1 post-discharge), intermediate (year 2–4 post-discharge), and late stage (year 5 post-discharge).

### 2.4. Statistical Analysis

Recording and computation of individual and Unit-average data during each patient’s hospitalisation were performed through an in-house software based on Microsoft Excel^®^ 2013 (Redmond, Washington, USA) and on a backbone algorithm developed for the collection of clinical information [38]. Univariate Cox regression was employed to identify potential predictors of overall post-discharge mortality. Survival was censored at the time of the last available information (extended follow-up). Multivariate Cox regression was run on the same dataset after exclusion of variables with no statistical significance at univariate analysis and redundant variables. Schoenfeld’s test was used to test the validity of the proportional hazard assumption. A chi-square test (with Fisher’s exact correction as appropriate) was used to assess the differential distribution of categorical variables among homogeneous timeframes. The Shapiro–Wilk test was performed to determine quantitative variable normality. The Mann–Whitney U-test was used to assess differences in quantitative variables among patients surviving or dying within each timeframe. Multivariate logistic regression was employed to dissect the independent contribution of significant variables at univariate analysis for mortality at each timeframe after exclusion of redundant variables. Composite clinimetrics, such as the CIRS and NEMS scores, were analysed in separate models. Statacorp STATA^®^ v15.1 (College Station, TX, USA) and Microsoft Excel^®^ 2019 (Redmond, Washington, DC, USA) were used for statistical analysis and data elaboration. Data are expressed as median (interquartile range, IQR) or number (percentage) unless otherwise specified.

## 3. Results

### 3.1. General Clinical Features

**Figure 1:**
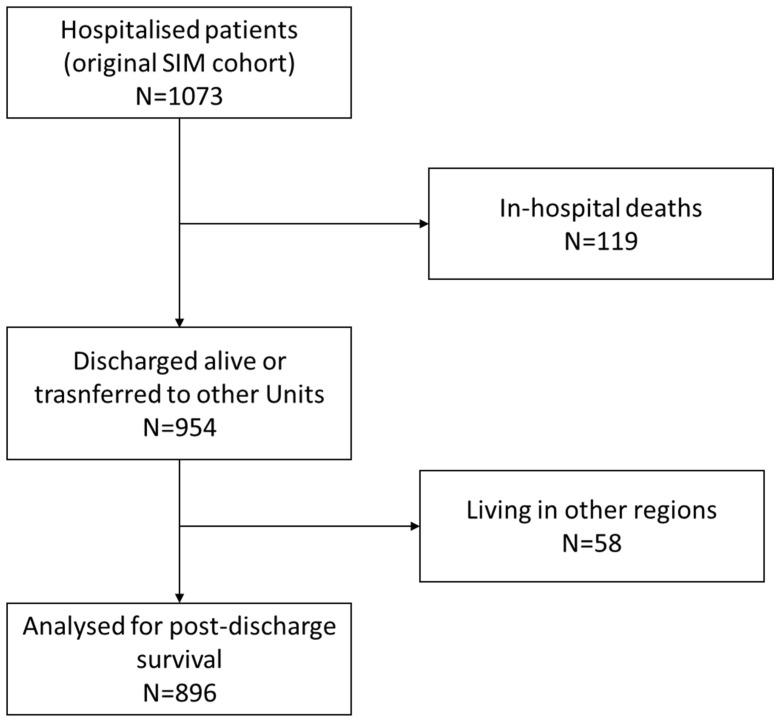
Study flow chart. This graph illustrates the number of patients included in this study as a subset of a cohort of patients who were hospitalised in an Internal Medicine Department (SIM cohort). These subjects (n = 1073) corresponded to the whole population of patients admitted during a 12-month observation window between 2016 and 2017. Among the 954 patients who were discharged alive, regional demographic data on survival were only available for 896, since 58 patients were living in other regions.

Of the 1073 subjects enrolled in the original prospective cohort of inpatients, 119 died during hospitalisation (11%), while 954 were either discharged alive or transferred to other wards (89%). Post-discharge follow-up data were available for 896 survivors and were used for analysis (Figure 1). Most subjects were men (520/896, 58%) with a median age of 74 (62–81) years. Almost all patients (92%) had more than one cause of morbidity. The most frequent comorbidities involved the cardiovascular (69%) and pulmonary (49%) domains, followed by metabolic (39%), neurological (38%), and renal (33%) disorders. A total of 470 (52%) discharged patients were frail (FI > 0.25). More than 1/3 of the patients were dependent for ADL/IADL. The characteristics of subjects analysed for their long-term outcome were consistent with those of the original cohort (Supplementary Table S2). The median NEMS was 18 (16–19). The median patient-to-senior physician ratio was 10 (9–11):1.

### 3.2. Post-Discharge Mortality

**Figure 2:**
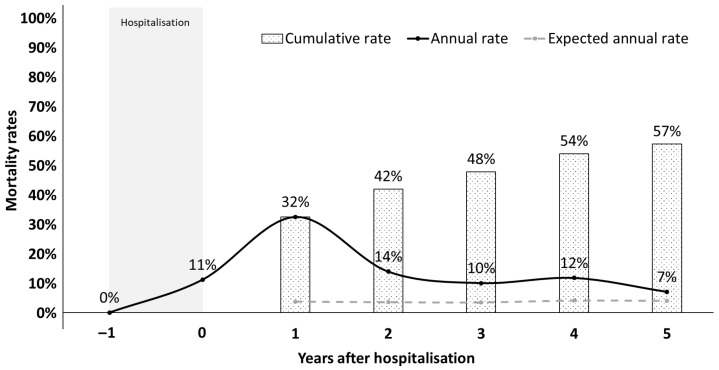
Post-discharge mortality. In this graph, mortality rates measured during (grey interval) and after hospitalisation are reported and compared with expected rates in the general population. Annual mortality rates calculated as the fraction of new deaths among survivors at each previous timepoint are depicted as a continuous line. Cumulative mortality rates calculated as the fraction of deaths since discharge (time zero) among patients included in the follow-up study (n = 896) are shown as bars. The grey dashed line depicts the expected annual mortality rates in the Italian general population. These rates were calculated based on the 2017–2022 mortality probabilities reported by the Italian National Institute of Statistics (ISTAT) and correspond to the mean of each age probability weighted on the frequency of each age in the study cohort year by year.

Of the 896 patients included in this study, 510 (57%) died within five years. Most of them (n = 290) died within the first post-discharge year, yielding a 32% mortality rate in the 12 months following hospitalisation. Within this timeframe, 50% of the patients died in the first three months, 26% between three and six months, and 24% between six and twelve months. Mortality rates during years 2–4 ranged between 10% and 14% of survivors in the previous year. At year five, we observed a mortality of 7% of survivors at year four, which was closer to the expected 3–4% annual mortality rate calculated for the Italian general population, adjusted for the age distribution observed in our cohort (Figure 2). Extended observation for further 10 (6–14) months showed that 24 additional deaths (6%) had occurred among survivors at five years.

Age, male sex, dependence on ADL/IADL, pulmonary, hepatic, upper gastrointestinal, metabolic, and neurological disorders, along with cancer, respiratory support with or without mechanical ventilation, and a lower number of patients with concomitant gastrointestinal disorders in the Unit at time of hospitalisation were independently associated with overall post-discharge mortality (Supplementary Table S3) based on multivariate Cox regression analysis. When NEMS and CIRS-SI were considered as surrogate markers of intensity of care and complexity and their components were excluded from the model, a significant association was found among mortality and higher NEMS and CIRS-SI scores, along with sex, age, dependence for ADL/IADL cancer, and the number of concomitant patients with upper gastrointestinal disorders (Supplementary Table S4). However, consistent with the observation of variable mortality rates over time, the proportional hazard assumption was rejected after performing the Schoenfeld’s test (χ^2^ = 77.93; *p* < 0.001).

### 3.3. Determinants of Early, Intermediate, and Late Post-Discharge Mortality

Based on the observation of unbalanced mortality rates and disproportional hazards over time, we hypothesised that different factors might have been associated with the risk of death at distinct stages. Therefore, we dissected the individual contribution of each clinical or healthcare-related feature recorded during hospitalisation to the annual mortality rates (Supplementary Tables S5 and S6) and confirmed that factors associated with early, intermediate, and late mortality were only partially overlapping (Table 1; Supplementary Figure S2).

#### 3.3.1. Univariate Analysis

Based on univariate analysis, early mortality was associated with higher age, male sex, dependence for ADL/IADL, cardiac, pulmonary, renal, hepatic, neurologic, and upper GI disorders, along with cancer, immunodepression, need for respiratory support, and higher CIRS and NEMS scores. Patients with immune-mediated disorders or exposure to Unit environments with a higher frequency of patient with immune-mediated disorders were inversely correlated with mortality at year 1. Conversely, an individual history of nosocomial infection or exposure to a Unit environment with more infected patients was associated with higher mortality rates within the first 12 months after discharge.

Patients deceasing within the intermediate stage of observation were also older than survivors and were more frequently males than females. Similar to patients with early mortality, patients dying within four years from discharge had a higher prevalence of cancer, cardiac, pulmonary, renal, and neurological disorders, a lower prevalence of immune-mediated disorders, and a higher prevalence of dependence for ADL/IADL. However, they did not show any significant association with nosocomial infections or liver disorders while demonstrating higher rates of arterial hypertension and of endocrine/metabolic disorders during hospitalisation. While higher NEMS and CIRS scores along with the need for oxygen support during hospitalisation remained strongly associated with death within year four, assistance-related variables changed significantly during the intermediate post-discharge timeframe, as infections and immune-mediated disorders in the hospitalisation environment no longer affected post-discharge mortality. In addition, mortality at years 2–4 was more frequent in patients with a lower number of exits from the Unit and exposed to Unit environments with lower numbers of patients with immune-mediated disorders.

Late mortality was associated with more advanced age but not with sex. An individual history of cardiac or upper GI disorders, along with higher NEMS or CIRS-CI scores and need for oxygen support without non-invasive mechanical ventilation, was more frequent in deceased patients than in survivors, while an individual history of immune-mediated diseases or exposure to Unit environments with higher frequencies of upper gastrointestinal disorders, genito-urinary tract disorders, renal disorders, or vascular/haematological disorders were protective for late mortality.

**Table 1:** Univariate associations with mortality by timeframe.

Variable	Total	Early (Year 1)		Intermediate (Year 2–4)		Late (Year 5)	
		Alive	Dead	Alive	Dead	Alive	Dead
	n = 896	n = 606	n = 290	n = 415	n = 191	n = 386	n = 29
Demographics and outcomes							
Age (years): median (IQR)	74 (62–81)	72 (57–80)	76 (69–83) ***	68 (52–77)	77 (71–84) ***	67 (51–77)	77 (74–80) ***
Sex (female): n (%)	376 (42)	271 (45)	105 (36) *	198 (48)	73 (38) *	185 (48)	13 (45)
Length of hospital stay(days): median (IQR)	12 (8–20)	12 (8–19)	13 (9–22)	12 (8–19)	12 (8–19)	12 (8–19)	12 (8–20)
Nosocomial infections during hospitalisation: n (%)	112 (13)	63 (10)	49 (17) **	40 (10)	23 (12)	35 (9)	5 (17)
Causes of morbidity: n (%)							
ADL/IADL dependence	322 (36)	169 (28)	153 (53) ***	101 (24)	68 (36) **	90 (23)	11 (38)
Cardiovascular disorders	622 (69)	408 (67)	214 (74) *	261 (63)	147 (77) **	234 (61)	27 (93) ***
Hypertension	481 (54)	312 (51)	169 (58)	201 (48)	111 (58) *	185 (48)	16 (55)
Cardiac disorders	469 (52)	303 (50)	166 (57) ***	175 (42)	128 (67) ***	154 (40)	21 (72) **
Lung disease	435 (49)	276 (46)	159 (55) **	173 (42)	103 (54) **	157 (41)	16 (55)
Kidney disease	294 (33)	178 (29)	116 (40) **	110 (27)	68 (36) *	102 (26)	8 (28)
Liver disease	135 (15)	77 (13)	58 (20) **	51 (12)	26 (14)	48 (12)	3 (10)
Cancer	276 (31)	132 (22)	144 (50) ***	73 (18)	59 (31) ***	67 (17)	6 (21)
End-stage cancer	52 (6)	11 (2)	41 (14) ***	7 (2)	4 (2)	7 (2)	0 (0)
Immune-mediated disorders	162 (18)	130 (21)	32 (11) ***	101 (24)	29 (15) *	99 (26)	2 (7) *
Endocrine-metabolic disorders	347 (39)	224 (37)	123 (42)	137 (33)	87 (46) **	124 (32)	13 (45)
Neurologic disorders/dementia	340 (38)	208 (34)	132 (46) **	128 (31)	80 (42) **	116 (30)	12 (41)
Psychiatric disorders	79 (9)	54 (9)	25 (9)	42 (10)	12 (6)	38 (10)	4 (14)
Vascular/hematologic disorders	307 (34)	54 (9)	26 (9)	142 (34)	69 (36)	136 (35)	6 (21)
Infectious disease	601 (67)	402 (66)	199 (69)	266 (64)	136 (71)	246 (64)	20 (69)
Upper GI disorders	72 (8)	40 (7)	32 (11) *	24 (6)	16 (8)	19 (5)	5 (17) *
Lower GI disorders	80 (9)	54 (9)	26 (9)	34 (8)	20 (10)	32 (8)	2 (7)
Genito-urinary disorders	98 (11)	67 (11)	31 (11)	41 (10)	26 (14)	39 (10)	2 (7)
Musculoskeletal/cutaneous disorders	185 (21)	131 (22)	54 (19)	93 (22)	38 (20)	87 (23)	6 (21)
Ocular/ENT disease	61 (7)	41 (7)	20 (7)	31 (7)	10 (5)	29 (8)	2 (7)
Immunodepression	191 (21)	114 (19)	77 (27) **	79 (19)	35 (18)	76 (20)	3 (10)
CIRS score: median (IQR)							
CIRS total score	9 (5–12)	8 (5–11)	10 (6–14) ***	7 (5–11)	9 (6–14) ***	7 (5–10)	9 (6–11)
CIRS severity score	0.6 (0.4–0.9)	0.6 (0.4–0.9)	0.8 (0.5–1.1) ***	0.5 (0.4–0.8)	0.7 (0.5–1.0) **	0.5 (0.3–0.8)	0.7 (0.5–0.9)
CIRS comorbidity score	3 (2–5)	3 (2–4)	4 (3–5) ***	3 (2–4)	4 (3–5) ***	3 (2–4)	4 (2–4) *
Intensity of care							
NEMS score: median (IQR)	18 (16–19)	18 (16–19)	18 (16–20) ***	17 (15–19)	18 (16–19) ***	17 (15–19)	19 (18–20) ***
Any respiratory support	478 (53)	296 (49)	182 (63) ***	174 (42)	122 (64) ***	153 (40)	21 (72) **
Oxygen without mechanical ventilation: n (%)	401 (45)	255 (42)	146 (50) *	153 (37)	102 (53) ***	136 (35)	17 (59) *
Mechanical ventilation: n (%)	83 (9)	44 (7)	39 (13) **	23 (6)	21 (11) *	19 (5)	4 (14)
Percentage of hospitalisation time with at least one exit for procedures: median (IQR)	13 (0–22)	13 (0–22)	13 (0–20)	15 (0–25)	11 (0–17) **	15 (0–25)	17 (0–25)
Ward-related variables (average): median (IQR)							
Infected patients (%)	72 (68–76)	71 (68–76)	72 (68–76) *	71 (67–75)	71 (68–76)	71 (67–75)	73 (69–75)
Patients with immune-mediated disorders (%)	17 (13–23)	18 (13–24)	16 (12–23) *	18 (13–24)	18 (13–24)	18 (12–24)	16 (13–22)
Patients with upper gastrointestinal tract disorders (%)	10 (7–14)	10 (7–14)	10 (6–14)	11 (7–15)	10 (6–13) *	11 (7–15)	8 (4–11) *
Patients with genito-urinary tract disorders (%)	10 (6–15)	9 (5–15)	11 (6–16)	10 (6–15)	9 (5–15)	10 (6–15)	8 (6–10) *
Patients with renal disorders (%)	29 (23–43)	30 (23–43)	29 (24–41)	30 (23–44)	29 (23–40)	30 (23–46)	26 (22–35) *
Patients with vascular/haematological disorders (%)	33 (29–42)	33 (29–42)	32 (29–41)	33 (29–42)	33 (29–43)	33 (29–43)	30 (28–34) *
Patients receiving oxygen without mechanical ventilation (%)	51 (40–55)	50 (39–55)	52 (41–55)	50 (39–55)	51 (40–56)	49 (39–55)	52 (41–57)
Mechanically ventilated patients (%)	12 (7–14)	11 (7–14)	12 (8–14)	11 (7–14)	11 (7–14)	11 (7–14)	12 (7–16)

Symbols: *: *p* < 0.05; **: *p* < 0.010, ***: *p* < 0.001. Abbreviations: ADL—activities of daily living, IADL—instrumental activities of daily living, CIRS—cumulative illness rating scale; NEMS—nine equivalents of nursing manpower score.

#### 3.3.2. Multivariate Analysis

Multivariate analysis showed that age was associated with mortality at any stage. Dependence for ADL/IADL, immunodepression, and renal disorders had a more prominent role for early mortality, while lung diseases along with the need for respiratory support during hospitalisation, liver diseases, and cancer impacted patient survival at early and intermediate stages. Patients who underwent a higher number of diagnostic or therapeutic procedures during hospitalisation were less likely to die at years 2–4. Late mortality was associated with cardiovascular and upper gastrointestinal disorders (Table 2). Relatively higher death rates were observed in frail patients compared to non-frail subjects at early stages. At later stages, high FI remained associated with death, although relatively higher mortality rates were observed in non-frail patients (Supplementary Table S7). In an alternative model including the NEMS and CIRS-SI scores instead of their components, higher CIRS-SI scores along with cancer were associated with short- and medium-term mortality, while higher NEMS scores predicted early or late mortality. Immunodepression and impaired ADL/IADL were selectively associated with early mortality, while age was confirmed as a transversal risk factor at any stage (Supplementary Table S8).

## 4. Discussion

In this study, we observed the long-term post-discharge trajectories of a population of patients hospitalised in an Internal Medicine Department over the course of one year. While confirming that patients admitted to Internal Medicine Departments are generally old, dependant for ADL/IADL, and frail, our data indicate that >50% of them die within five years. Mortality peaked within the first 3–12 months, suggesting that, irrespective of cancer diagnosis, up to 1/3 of Internal Medicine Department patients might require palliative care and possibly be more easily identified based on age, history of cancer, immunodepression, dependence for ADL/IADL or neurological disorders, need for respiratory support during hospitalisation and/or lung, liver, or kidney morbidity, in line with reports on selected patient groups [32,39,40,41,42]. After year one post-discharge, patient mortality was reduced, although it was still significantly higher than in the general population and affected by the intensity of care during hospitalisation besides age, lung and liver morbidity, cancer, and neurological disorders. After year four, patients’ mortality approached that of the general population and was affected more strongly by age and gastrointestinal and cardiovascular morbidity, with a relatively less significant role of cancer and other comorbidities.

Predicted mortality within 12 months has been conventionally set as the prognostic threshold for palliative care [43,44]. Estimates based on retrospective analyses of annual mortality, hospital discharges, and demographics of patients participating in palliative care programmes anticipate that 2–8% of the elderly general population would annually be eligible for palliative care [8,43,45,46]. Complementary to this information, our data indicate that up to one third of patients hospitalised in Internal Medicine Departments could require palliative care due to a higher prevalence of frailty and likely die within three to twelve months after discharge. These results are consistent with previous works exploring short- [32,39,40,41,42] and medium-term [47,48,49] post-discharge mortality and consolidate them by including more robust data from a large-sized cohort corresponding to the whole population of patients discharged from an Internal Medicine Department throughout a year. Thanks to the number of patients included in our analysis, we were also able to dissect potential predictors of early mortality beyond generic descriptors such as age and sex. Neurological disorders, possibly in association with impaired ADL/IADL, were particularly relevant at this stage, in line with their high prevalence among palliative care beneficiaries [43]. Renal diseases and immunodepression were also selectively associated with early mortality in our cohort, while they had a lower impact on longer-term mortality risk. Impaired renal function is a major cause of disability in the elderly and a major predictor of mortality in patients with neurological and liver disorders [50,51,52]. Furthermore, liver and renal failures might synergise with the toxic effects of drugs, especially in patients with polypharmacy and impaired cognition [53] and in the early post-discharge phase, when extended treatment of acute conditions might increase the number medications [54]. Consistent with these data, recent research from the BECLAP and the REPOSI study groups identified kidney function compromise as a major determinant of death at three months, along with male sex, bedridden status, recent hospital admissions, dysphagia, severe dementia, and hypoalbuminemia [41,42]. Immunodepression is relatively common in hospitalised patients and could have enhanced the detrimental impact of other causes of morbidity by increasing the likelihood of infections [55,56,57]. Taken together, these results suggest that hospitalised patients with renal failure, immunodepression, and/or dependence for ADL/IADL along with other potential predictors of early mortality might be prioritised to palliative care [41,42].

Our analysis of post-discharge mortality extended beyond the first twelve months following hospitalisation. Previous studies in heterogeneous sets of inpatients and outpatients with multiple comorbidities [48,49] showed that active cancer, neurological compromise, and loss of autonomy (especially with insufficient caregiver support), synergise with recurrent hospitalisations and determine medium-term survival. Our results complement this evidence, showing that in a well-defined population of Internal Medicine Department patients, neurological morbidity, lung disorders and/or respiratory insufficiency, liver disorders, and cancer remain major predictors of mortality at years 2–4, while mortality rates drop to less than half the rate in the first year, possibly providing clues for identifying candidates for simultaneous palliative care [21,27,58,59,60].

Starting from the fifth year from hospitalisation, mortality rates further decreased, along with changes in potential predictors of death. Cardiovascular morbidity and upper gastrointestinal disorders were more significantly associated with late rather than early mortality in our cohort. These data are consistent with the primacy of cardiovascular diseases among the causes of death in the European elderly population and with the relative decline in deaths due to cancer in this advanced age group [61]. Gastrointestinal disorders constitute a proxy of frailty, with population-level studies revealing a high risk of mortality, often due to causes other than gastrointestinal complications [62] including drug-related toxicities [63,64]. In our cohort, upper gastrointestinal disorders had a low prevalence (less than 10%) but were highly associated with late-stage mortality. Almost 3/4 of the subjects died within five years after discharge, and 20% of patients dying after year four had upper gastrointestinal morbidity. Paradoxically, higher survival rates were observed in subjects hospitalised together with patients with upper gastrointestinal disorders because they lacked that predictor of mortality.

Taken together, our data suggest that patient features during hospitalisation can predict post-discharge survival and show a differential impact in early and late stages. Nonetheless, these conclusions should be critically interpreted considering the limitations of this study. Our single-centre design did not address clinical and healthcare variability among different regions and institutions, which constitutes a matter of high interest for future studies. In the Italian healthcare context, despite substantial homogeneity in hospital standards and practices, progressive withdrawal of public commitment to welfare policies and aggressive healthcare governance decentralisation has exacerbated existing sociodemographic inequalities and vulnerabilities. The most affected parameter has been free healthcare accessibility, with poorer regions underpowering available services due to the need to contain budget deficits, wealthier regions having to face increased demands due to within-country migrations, and policies that intend individual/local wealth protection as a priority reducing the availability of diffuse public healthcare services while overwhelming hospital hubs [65,66,67]. The Region of Lombardy, where our study was conducted, currently faces an unresolved problem of excessive depotentiation of primary healthcare [68], and our high rates of post-discharge mortality might at least in part have been affected by this vulnerability. In addition, lack of information about patient-specific causes of death prevented deeper deductions regarding the mechanisms driving mortality in our cohort. This could have been particularly relevant for the intermediate timeframe of observation, which included the peak of the recent COVID-19 pandemic and might have introduced a bias in terms of susceptibility to death in patients with pre-existing chronic disorders, including respiratory insufficiency [69,70,71]. A milder additional bias might have been introduced in terms of death rates calculated after 3–4 years after discharge due to the slight but significant increase in deaths having occurred in 2020, especially in Lombardy [72]. Data on determinants of frailty such as nutritional status would also have disclosed additional hints to define at-risk subgroups. We also did not record data on patients’ medication number and type and were thus unable to analyse potential associations between death and specific treatments. Further studies collecting extensive patient-level information from hospital admission to long-term primary care follow up would be needed to precisely dissect how baseline individual variables, eventual accrual of morbidity, and potential confounders contribute to post-discharge mortality at distinct timepoints. This work was focused on mortality only. Further studies including data on re-admissions and changes in quality of life are warranted to better define the profile of patients in need of early palliative care [40]. This would be particularly relevant to define pertinent indications to simultaneous palliative care. In fact, while our data indicate that patients sufficiently fit to undergo diagnostic/therapeutic procedures had longer lifespans, we were unable to assess whether this was associated with acceptable levels of wellbeing. To reduce the risks of bias due to the violation of the proportional hazard assumption with unstable mortality rates over time, we chose to conduct separate analyses of distinct timeframes. This approach is not devoid of potential limitations, since it might have oversimplified the sets of variables associated with adverse outcomes in a given timeframe. Yet, it enabled a more accurate identification of potential factors more strongly associated with mortality in the long-term, thus complementing existing literature [48,49]. Limited information about patients’ social and support context might have reduced our ability to identify factors potentially counterbalancing intrinsic determinants of reduced survival. Conversely, data on potential aggravating factors such as a poor nutritional status and/or exposure to polypharmacy would have enabled a more refined definition of at-risk subgroups [73]. In the absence of appropriate measures for intensity of care in non-intensive care unit settings, we relied on the NEMS to maximise result reproducibility and assess the role of the procedures (such as the use of vasoactive drugs and of mechanical ventilation) that are routinely performed in the study unit and constitute proxies of intensive care according to the NEMS definitions. Developing intensity of care measures specifically fit for Internal Medicine Departments remains a fascinating prospect for the future.

Notwithstanding these limitations, this study has the strength of involving a compact cohort constituted by all patients discharged alive from an Internal Medicine Department during a year and residing in the same region. Furthermore, it builds on a relatively comprehensive set of variables recorded daily at an individual patient level to record as faithfully as possible all aspects of complexity characterising patients admitted in general Internal Medicine wards. Larger multicentre studies are still warranted to further consolidate our findings.

## 5. Conclusions

Patients hospitalised in Internal Medicine Departments are characterised by advanced age, multi-morbidity, and high rates of post-discharge mortality, with a peak during the first 12 months. While age remains a transversal determinant of post-discharge mortality, renal disorders along with immunodepression and dependence for ADL/IADL are specifically associated with early mortality and might synergise with risk factors such as liver disorders, cancer, neurological disorders, disability, and respiratory failure, which are also relevant for medium-term mortality. Cardiovascular and upper gastrointestinal disorders share a strong association with late mortality despite differing in prevalence. Accurate dissection of patient early-, medium-, and long-term risk based on these data might pave the way for personalised strategies of care for CCF patients, including early access to palliative care or simultaneous palliative care programmes.

## Figures and Tables

**Table 2:** Associations with mortality by timeframe based on multivariate logistic regression.

Variable	Early (Year 1)		Intermediate (Year 2–4)		Late (Year 5)	
	*p*	RR (95% CI)	*p*	RR (95% CI)	*p*	RR (95% CI)
Demographics						
Age	<0.001	1.04 (1.03–1.06)	<0.001	1.06 (1.04–1.08)	0.008	1.05 (1.01–1.09)
Sex	0.071	-	0.120	-	0.673	-
Nosocomial infections during hospitalisation	0.689	-	0.465	-	0.923	-
Causes of morbidity						
ADL/IADL dependence	0.001	1.99 (1.31–3.03)	0.706	-	0.939	-
Cardiovascular disorders	0.569	-	0.530	-	0.034	5.23 (1.13–24.16)
Lung disease	0.008	1.72 (1.15–2.58)	0.036	1.58 (1.03–2.42)	0.073	-
Liver disease	<0.001	2.69 (1.59–4.53)	0.030	1.91 (1.06–3.43)	0.582	-
Kidney disease	0.017	1.70 (1.10–2.63)	0.436	-	0.973	-
Cancer	<0.001	5.51 (3.55–8.54)	<0.001	2.89 (1.78–4.68)	0.269	-
Immune-mediated disorders	0.107	-	0.937	-	0.310	-
Endocrine-metabolic disorders	0.202	-	0.076	-	0.609	-
Neurologic disorders/dementia	0.010	1.71 (1.14–2.58)	0.039	1.58 (1.02–2.45)	0.535	-
Upper GI disorders	0.153	-	0.138	-	0.005	5.96 (1.69–20.98)
Immunodepression	0.004	2.13 (1.26–3.58)	0.215	-	0.946	-
Intensity of care						-
Any respiratory support	0.032	1.56 (1.04–2.34)	0.016	1.7 (1.1–2.61)	0.085	-
Percentage of hospitalisation time with at least one exit for procedures	0.089	-	0.001	0.08 (0.02–0.35)	0.683	-
Ward-related variables (average)						
Infected patients	0.376	-	0.502	-	0.142	-
Patients with immune-mediated disorders	0.414	-	0.840	-	0.574	-
Patients with upper gastrointestinal tract disorders	0.334	-	0.112	-	0.324	-
Patients with genito-urinary tract disorders	0.850	-	0.460	-	0.583	-
Patients with renal disorders	0.182	-	0.360	-	0.529	-
Patients with vascular/haematological disorders	0.695	-	0.454	-	0.267	-
Constant	0.002	0.01 (0–0.17)	<0.001	0 (0–0.07)	0.005	0 (0–0.04)

Abbreviations: ADL—activities of daily living, IADL—instrumental activities of daily living.

## Data Availability

The data will be made available upon request due to restrictions (not included in the original protocol).

## Supplementary Materials

The following supporting information can be downloaded at: https://www.mdpi.com/article/10.3390/jcm15020850/s1, Figure S1: Schematic representation of individual and Unit variables; Figure S2: heatmap of the strength of association among death and individual clinical and healthcare-related factors (univariate analysis); Table S1: components of the Frailty Index (FI); Table S2: clinical characteristics of the original and follow-up cohort; Table S3: factors associating with overall post-discharge mortality; Table S4: alternative model for factors associating with overall post-discharge mortality; Table S5: factors associating with mortality by year; Table S6: annual mortality rates by categorical factors; Table S7: Deaths among frail and non-frail patients; Table S8: alternative model for associations with mortality by timeframe at multivariate logistic regression.
