# Supplementary material for: Variability in Post-Discharge Mortality Rates and Predictors over Time: Data from a Five-Year Ward-Wide Study
Source: J Clin Med. 2026 Jan 20;15(2):850. doi: 10.3390/jcm15020850 (PMC12841896; doi:10.3390/jcm15020850)
Supplement: Supplementary file 1 [file jcm-15-00850-s001.zip › jcm-4059285-supplementary.pdf]

Supplemental Material

to

Variability in post-discharge mortality rates and predictors over time: data from a five-year ward-wide study

Giuseppe A. Ramirez[1,2], Bruno N. Germinario[3], Giovanni Benanti[1,3], Pier Francesco Caruso[2,3], Francesca Mette[2,3], Gaia Pagliula[2,3], Adriana Cariddi[1,2], Silvia Sartorelli[2,3], Elisabetta Falbo[2,3], Alessandro Marinosci[3], Francesca Farina[2,3], Giacomo Pacioni[2,3], Elena Rela[2,3], Pietro Barbieri[4], Moreno Tresoldi[3], and Enrica P. Bozzolo[3]

1) Unit of Immunology, Rheumatology, Allergy and Rare Diseases, IRCCS Ospedale San Raffaele, Milan, Italy
2) Università Vita-Salute San Raffaele, Milan, Italy
3) Unit of General Medicine and Advanced Care, IRCCS Ospedale San Raffaele, Milan, Italy
4) Clinical Management Unit, IRCCS Ospedale San Raffaele, Milan, Italy

Supplemental Table S1: components of the Frailty Index (FI)

ITEMS	CUT-OFF
Cardiological disease	1=present 0=absent
Lung disease	1=present 0=absent
Kidney disease	1=present 0=absent
Neurological disease	1=present 0=absent
Psychiatric disease	1=present 0=absent
Oftalmological and ENT disease	1=present 0=absent
Endocrine and Metabolic disease	1=present 0=absent
Liver disease	1=present 0=absent
Upper GI disease	1=present 0=absent
Lower GI disease	1=present 0=absent
Genito-urinary disease	1=present 0=absent
Rheumatological disease	1=present 0=absent
Haematological disease	1=present 0=absent
Immunedeppresed disease	1=present 0=absent
Active Infectious disease	1=present 0=absent
Active Oncological disease	1=present 0=absent
NEMS	18
CIRS	9
Need of O2 therapy	1=present 0=absent
Dependendence on ADL	1=present 0=absent

The index was composed of 20 items following the criteria by Theou et al., Age Ageing, 2023. These criteria encompassed clinical history of chronic cardiovascular disorders, lung disorders, renal disorders, neurological disorders, psychiatric disorders, ocular and ENT disorders, endometabolic disorders, hepatic disorders, upper GI disorders, Lower Gi disorders urological disorders, rheumatological disorders, haematological disorders, immunosuppression, active infectious disorders, active cancer, dementia, a CIRS and a NEMS value over the median value of the study population, needs of O2 therapy, dependence on ADL/IADL.

Supplemental Table S2: clinical characteristics of the original and follow-up cohort

	Original cohort (N=1,073)	Follow-up cohort (N=896)
**Demographics and outcomes**		
Women: n (%)	445 (41)	376 (42)
Age: median (IQR)	74 (62-82)	74 (62-81)
In-hospital deaths: n (%)	119 (11)	0 (0)
Nosocomial infections: n (%)	136 (13)	112 (13)
Time to discharge (days): median (IQR)	12 (8-20)	12 (8-20)
**Morbidity**		
ADL/IADL dependence: n (%)	436 (41)	322 (36)
Cardiovascular disorders: n (%)	745 (69)	622 (69)
Cardiac disorders: n (%)	566 (53)	469 (52)
Hypertension: n (%)	583 (54)	481 (54)
Pulmonary disorders: n (%)	517 (48)	435 (49)
Renal disorders: n (%)	362 (34)	294 (33)
Upper gastrointestinal tract disorders: n (%)	103 (10)	72 (8)
Lower gastrointestinal tract disorders: n (%)	105 (10)	80 (9)
Liver disorders: n (%)	170 (16)	135 (15)
Metabolic disorders: n (%)	425 (40)	347 (39)
Immune-mediated disorders: n (%)	180 (17)	162 (18)
Neoplastic disorders: n (%)	350 (33)	276 (31)
End-stage neoplastic disorders: n (%)	76 (7)	52 (6)
Neurological disorders: n (%)	439 (41)	340 (38)
Psychiatric disorders: n (%)	100 (9)	79 (9)
Immunocompromised subjects: n (%)	226 (21)	191 (21)
CIRS total score: median (IQR)	9 (6-13)	9 (5-12)
CIRS severity score: median (IQR)	0.7 (0.4-0.9)	0.6 (0.4-1.0)
CIRS comorbidity score: median (IQR)	3.0 (2.0-5.0)	3.0 (2.0-5.0)
**Intensity of care**		
Continuous vital signs monitoring*: n (%)	1,045 (97)	873 (97)
Any oxygen support: n (%)	589 (55)	478 (53)
Nasal cannulas or Venturi's mask: n (%)	488 (45)	401 (45)
Non-invasive ventilation: n (%)	109 (10)	83 (9)
Intravenous treatments: n (%)	999 (93)	830 (93)
Vasoactive circulation support with one drug at least once: n (%)	17 (2)	10 (1)
Vasoactive circulation support with more than one drug at least once: n (%)	0 (0)	0 (0)
Dialysis at least once: n (%)	14 (1)	13 (1)
At least one in-ward non-standard procedure: n(%)	177 (16)	126 (14)
At least one exit from the ward for other procedures: n(%)	760 (71)	631 (70)
Patients requiring surgery: n(%)	27 (3)	17 (2)
NEMS average score: median (IQR)	18 (16-19)	18 (16-19)

Abbreviations
ADL/IADL: general or instrumental activities of daily living. CIRS: cumulative illness rating scale. NEMS: nine equivalents of manpower score

Supplemental Table S3: factors associating with overall post-discharge mortality

Variable	Total<br><br>N=896	Alive at follow up end<br><br>N=362	Dead at follow-up end<br><br>N=534	Univariate	Multivariate	HR (95%CI)
**Demographics and outcomes**						
Age (years): median (IQR)	74 (62-81)	65 (51-75)	77 (70-83)	<0.001	<0.001	1.03 (1.02 - 1.04)
Sex (female): N(%)	376 (42)	176 (49)	200 (37)	0.001	0.006	0.77 (0.64 - 0.93)
Length of hospital stay(days): median (IQR)	12 (8-20)	12 (8-19)	12 (8-21)	<0.001	NS	
Nosocomial infections during hospitalisation: N(%)	112 (13)	32 (9)	80 (15)	0.001	NS	
**Causes of morbidity**: N(%)						
ADL/IADL dependence	322 (36)	83 (23)	239 (45)	<0.001	0.012	1.30 (1.06-1.59)
Cardiovascular disorders	622 (69)	215 (59)	407 (76)	<0.001	NM[2]	
Hypertension	481 (54)	168 (46)	313 (59)	<0.001	NS	
Cardiac disorders	469 (52)	139 (38)	330 (62)	<0.001	NS	
Lung disease	435 (49)	150 (41)	285 (53)	<0.001	0.030	1.34 (1.02-1.51)
Kidney disease	294 (33)	92 (25)	202 (38)	<0.001	NS	
Liver disease	135 (15)	48 (13)	87 (16)	0.033	0.001	1.51 (1.18-1.92)
Cancer	276 (31)	64 (18)	212 (40)	<0.001	<0.001	2.32 (1.93-2.79)
End-stage cancer	52 (6)	7 (2)	45 (8)	<0.001	NM[2]	
Immune-mediated disorders	162 (18)	94 (26)	68 (13)	<0.001	NS	
Endocrine-metabolic disorders	347 (39)	111 (31)	236 (44)	<0.001	0.037	1.23 (1.01-1.49)
Neurologic disorders / dementia	340 (38)	111 (31)	229 (43)	<0.001	0.007	1.30 (1.07-1.57)
Psychiatric disorders	79 (9)	35 (10)	44 (8)	NS	NM[1]	
Vascular / hematologic disorders	307 (34)	131 (36)	176 (33)	NS	NM[1]	
Infectious disease	601 (67)	225 (62)	376 (70)	0.027	NS	
Upper GI disorders	72 (8)	18 (5)	54 (10)	0.001	0.009	1.48 (1.10-1.98)
Lower GI disorders	80 (9)	30 (8)	50 (9)	NS	NM[1]	
Genito-urinary disorders	98 (11)	37 (10)	61 (11)	NS	NM[1]	
Musculoskeletal/cutaneous disorders	185 (21)	80 (22)	105 (20)	NS	NM[1]	
Ocular / ENT disease	61 (7)	28 (8)	33 (6)	NS	NM[1]	

**CIRS score**: median (IQR)						
CIRS total score	9 (5-12)	7 (5-10)	9 (6-14)	<0.001	NM2	
CIRS severity score	0.6 (0.4-0.9)	0.5 (0.3-0.8)	0.7 (0.5-1.0)	<0.001	NM2	
CIRS comorbidity score	3 (2-5)	3 (2-4)	4 (2-5)	<0.001	NM2	
**Intensity of care**						
NEMS score: median (IQR)	18 (16-19)	17 (15-19)	18 (16-19)	<0.001	NM2	
Any respiratory support	478 (53)	138 (38)	340 (64)	<0.001	NM2	
Oxygen without mechanical ventilation: N(%)	401 (45)	121 (33)	280 (52)	<0.001	0.023	1.26 (1.03-1.54)
Mechanical ventilation: N(%)	83 (9)	19 (5)	64 (12)	<0.001	0.010	1.51 (1.10-2.07)
Percentage of hospitalisation time with at least one exit for procedures: median (IQR)	13 (0-22)	15 (1-25)	11 (0-20)	<0.001	NS	
Unit-related variables (average): median (IQR)						
Patient age (years)	71 (69-72)	70 (69-72)	71 (70-72)	0.004	NS	
Infected patients (%)	72 (68-76)	71 (67-75)	72 (68-76)	0.005	NS	
Patients with immune-mediated disorders (%)	17 (13-23)	18 (12-24)	17 (13-23)	0.027	NS	
Patients with upper gastrointestinal tract disorders (%)	10 (7-14)	11 (7-16)	10 (6-14)	0.019	0.007	0.08 (0.01-0.50)
Patients receiving oxygen without mechanical ventilation (%)	51 (40-55)	49 (39-55)	52 (41-56)	0.046	NS	
Mechanically ventilated patients (%)	12 (7-14)	11 (7-14)	12 (7-14)	NS	NM1	

Abbreviations and symbols

ADL – activities of daily living. CI: confidence interval. CIRS – cumulative illness rating scale. HR: hazard ratio. IADL – instrumental activities of daily living. NEMS – nine equivalents of nursing manpower score. NM: not included in the model for 1 no association at univariate analysis, 2 redundance with other variables. NS: not statistically significant.

Supplemental Table S4: alternative model for factors associating with overall post-discharge mortality

Variable	Total	Alive at follow up end	Dead at follow up end	Univariate	Multivariate	HR (95%CI)
	N=896	N=362	N=534			
**Demographics and outcomes**						
Age (years): median (IQR)	74 (62-81)	65 (51-75)	77 (70-83)	<0.001	<0.001	1.03 (1.02 - 1.04)
Sex (female): N(%)	376 (42)	176 (49)	200 (37)	0.001	0.006	0.77 (0.65 - 0.93)
Length of hospital stay(days): median (IQR)	12 (8-20)	12 (8-19)	12 (8-21)	<0.001	NS	
Nosocomial infections during hospitalisation: N (%)	112 (13)	32 (9)	80 (15)	0.001	NS	
**Causes of morbidity**: N(%)						
ADL/IADL dependence	322 (36)	83 (23)	239 (45)	<0.001	0.011	1.31 (1.07-1.61)
Cardiovascular disorders	622 (69)	215 (59)	407 (76)	<0.001	NM[2]	
Hypertension	481 (54)	168 (46)	313 (59)	<0.001	NM[2]	
Cardiac disorders	469 (52)	139 (38)	330 (62)	<0.001	NM[2]	
Lung disease	435 (49)	150 (41)	285 (53)	<0.001	NM[2]	
Kidney disease	294 (33)	92 (25)	202 (38)	<0.001	NM[2]	
Liver disease	135 (15)	48 (13)	87 (16)	0.033	NM[2]	
Cancer	276 (31)	64 (18)	212 (40)	<0.001	<0.001	2.17 (1.81-2.59)
End-stage cancer	52 (6)	7 (2)	45 (8)	<0.001	NM[2]	
Immune-mediated disorders	162 (18)	94 (26)	68 (13)	<0.001	NS	
Endocrine-metabolic disorders	347 (39)	111 (31)	236 (44)	<0.001	NM[2]	
Neurologic disorders / dementia	340 (38)	111 (31)	229 (43)	<0.001	NM[2]	
Psychiatric disorders	79 (9)	35 (10)	44 (8)	NS	NM[2]	
Vascular / hematologic disorders	307 (34)	131 (36)	176 (33)	NS	NM[2]	
Infectious disease	601 (67)	225 (62)	376 (70)	0.027	NS	
Upper GI disorders	72 (8)	18 (5)	54 (10)	0.001	NM[2]	
Lower GI disorders	80 (9)	30 (8)	50 (9)	NS	NM[1]	
Genito-urinary disorders	98 (11)	37 (10)	61 (11)	NS	NM[1]	
Musculoskeletal/cutaneous disorders	185 (21)	80 (22)	105 (20)	NS	NM[1]	
Ocular / ENT disease	61 (7)	28 (8)	33 (6)	NS	NM[1]	
**CIRS score**: median (IQR)						
CIRS total score	9 (5-12)	7 (5-10)	9 (6-14)	<0.001	NM[2]	
CIRS severity score	0.6 (0.4-0.9)	0.5 (0.3-0.8)	0.7 (0.5-1.0)	<0.001	<0.001	1.76 (1.34-2.30)
CIRS comorbidity score	3 (2-5)	3 (2-4)	4 (2-5)	<0.001	NM[2]	
**Intensity of care**						
NEMS score: median (IQR)	18 (16-19)	17 (15-19)	18 (16-19)	<0.001	0.001	1.04 (1.01-1.6)
Any respiratory support	478 (53)	138 (38)	340 (64)	<0.001	NM[2]	
Oxygen without mechanical ventilation: N(%)	401 (45)	121 (33)	280 (52)	<0.001	NM[2]	
Mechanical ventilation: N(%)	83 (9)	19 (5)	64 (12)	<0.001	NM[2]	
Percentage of hospitalisation time with at least one exit for procedures: median (IQR)	13 (0-22)	15 (1-25)	11 (0-20)	<0.001	NS	
Unit-related variables (average): median (IQR)						
Patient age (years)	71 (69-72)	70 (69-72)	71 (70-72)	0.004	NS	
Infected patients (%)	72 (68-76)	71 (67-75)	72 (68-76)	0.005	NS	
Patients with immune-mediated disorders (%)	17 (13-23)	18 (12-24)	17 (13-23)	0.027	NS	
Patients with upper gastrointestinal tract disorders (%)	10 (7-14)	11 (7-16)	10 (6-14)	0.019	0.010	0.09 (0.01-0.56)
Patients receiving oxygen without mechanical ventilation (%)	51 (40-55)	49 (39-55)	52 (41-56)	0.046	NS	
Mechanically ventilated patients (%)	12 (7-14)	11 (7-14)	12 (7-14)	NS	NM[1]	

Abbreviations and symbols
CI: confidence interval. HR: hazard ratio. NM: not included in the model for 1 no association at univariate analysis, 2 redundance with other variables. NS: not statistically significant.

Supplemental Table S5: factors associating with mortality by year

Variable	Total	Year 1		Year 2		Year 3		Year 4		Year 5	
		Alive	Dead	Alive	Dead	Alive	Dead	Alive	Dead	Alive	Dead
	N=896	N=606	N=290	N=522	N=84	N=470	N=52	N=415	N=55	N=386	N=29
Demographics and outcomes											
Age (years): median (IQR)	74 (62-81)	72 (57-80)	76 (69-83)	71 (55-78)	78 (72-84)	69 (54-78)	76 (69-84)	68 (52-77)	77 (71-82)	67 (51-77)	77 (74-80)
Sex (female): N(%)	376 (42)	271 (45)	105 (36)	241 (46)	30 (36)	220 (47)	21 (40)	198 (48)	22 (40)	185 (48)	13 (45)
Length of hospital stay(days): median (IQR)	12 (8-20)	12 (8-19)	13 (9-22)	12 (8-19)	12 (9-18)	12 (8-19)	11 (8-22)	12 (8-19)	10 (8-18)	12 (8-19)	12 (8-20)
Nosocomial infections during hospitalisation: N (%)	112 (13)	63 (10)	49 (17)	55 (11)	8 (10)	46 (10)	9 (17)	40 (10)	6 (11)	35 (9)	5 (17)
Causes of morbidity: N(%)											
ADL/IADL dependence	322 (36)	169 (28)	153 (53)	129 (25)	40 (48)	114 (24)	15 (29)	101 (24)	13 (24)	90 (23)	11 (38)
Cardiovascular disorders	622 (69)	408 (67)	214 (74)	343 (66)	65 (77)	302 (64)	41 (79)	261 (63)	41 (75)	234 (61)	27 (93)
Hypertension	481 (54)	312 (51)	169 (58)	264 (51)	48 (57)	230 (49)	34 (65)	201 (48)	29 (53)	185 (48)	16 (55)
Cardiac disorders	469 (52)	303 (50)	166 (57)	245 (47)	58 (69)	210 (45)	35 (67)	175 (42)	35 (64)	154 (40)	21 (72)
Lung disease	435 (49)	276 (46)	159 (55)	229 (44)	47 (56)	202 (43)	27 (52)	173 (42)	29 (53)	157 (41)	16 (55)
Kidney disease	294 (33)	178 (29)	116 (40)	153 (29)	25 (30)	131 (28)	22 (42)	110 (27)	21 (38)	102 (26)	8 (28)
Liver disease	135 (15)	77 (13)	58 (20)	66 (13)	11 (13)	60 (13)	6 (12)	51 (12)	9 (16)	48 (12)	3 (10)
Cancer	276 (31)	132 (22)	144 (50)	107 (20)	25 (30)	84 (18)	23 (44)	73 (18)	11 (20)	67 (17)	6 (21)
End-stage cancer	52 (6)	11 (2)	41 (14)	8 (2)	3 (4)	8 (2)	0 (0)	7 (2)	1 (2)	7 (2)	0 (0)
Immune-mediated disorders	162 (18)	130 (21)	32 (11)	122 (23)	8 (10)	108 (23)	14 (27)	101 (24)	7 (13)	99 (26)	2 (7)
Endocrine-metabolic disorders	347 (39)	224 (37)	123 (42)	189 (36)	35 (42)	164 (35)	25 (48)	137 (33)	27 (49)	124 (32)	13 (45)
Neurologic disorders / dementia	340 (38)	208 (34)	132 (46)	165 (32)	43 (51)	145 (31)	20 (38)	128 (31)	17 (31)	116 (30)	12 (41)
Psychiatric disorders	79 (9)	54 (9)	25 (9)	48 (9)	6 (7)	45 (10)	3 (6)	42 (10)	3 (5)	38 (10)	4 (14)
Vascular / hematologic disorders	307 (34)	54 (9)	26 (9)	179 (34)	32 (38)	162 (34)	17 (33)	142 (34)	20 (36)	136 (35)	6 (21)
Infectious disease	601 (67)	402 (66)	199 (69)	350 (67)	52 (62)	308 (66)	42 (81)	266 (64)	42 (76)	246 (64)	20 (69)
Upper GI disorders	72 (8)	40 (7)	32 (11)	32 (6)	8 (10)	29 (6)	3 (6)	24 (6)	5 (9)	19 (5)	5 (17)
Lower GI disorders	80 (9)	54 (9)	26 (9)	46 (9)	8 (10)	38 (8)	8 (15)	34 (8)	4 (7)	32 (8)	2 (7)
Genito-urinary disorders	98 (11)	67 (11)	31 (11)	57 (11)	10 (12)	47 (10)	10 (19)	41 (10)	6 (11)	39 (10)	2 (7)
Musculoskeletal/cutaneous disorders	185 (21)	131 (22)	54 (19)	112 (21)	19 (23)	104 (22)	8 (15)	93 (22)	11 (20)	87 (23)	6 (21)
Ocular / ENT disease	61 (7)	41 (7)	20 (7)	39 (7)	2 (2)	35 (7)	4 (8)	31 (7)	4 (7)	29 (8)	2 (7)

Immunodepression: N(%)	191 (21)	114 (19)	77 (27)	105 (20)	9 (11)	90 (19)	15 (29)	79 (19)	11 (20)	76 (20)	3 (10)
CIRS score: median (IQR)											
CIRS total score	9 (5-12)	8 (5-11)	10 (6-14)	8 (5-11)	9 (7-13)	8 (5-11)	9 (6-15)	7 (5-11)	9 (6-14)	7 (5-10)	9 (6-11)
CIRS severity score	0,6 (0,4-0,9)	0,6 (0,4-0,9)	0,8 (0,5-1,1)	0,6 (0,4-0,9)	0,7 (0,5-0,9)	0,5 (0,4-0,8)	0,7 (0,5-1,2)	0,5 (0,4-0,8)	0,6 (0,4-1,1)	0,5 (0,3-0,8)	0,7 (0,5-0,9)
CIRS comorbidity score	3 (2-5)	3 (2-4)	4 (3-5)	3 (2-4)	4 (3-5)	3 (2-4)	4 (2-6)	3 (2-4)	3 (2-6)	3 (2-4)	4 (2-4)
Intensity of care											
NEMS score: median (IQR)	18 (16-19)	18 (16-19)	18 (16-20)	17 (15-19)	18 (17-19)	17 (15-19)	18 (16-19)	17 (15-19)	18 (15-19)	17 (15-19)	19 (18-20)
Any respiratory support	478 (53)	296 (49)	182 (63)	238 (46)	58 (69)	203 (43)	35 (67)	174 (42)	29 (53)	153 (40)	21 (72)
Oxygen without mechanical ventilation: N(%)	401 (45)	255 (42)	146 (50)	207 (40)	48 (57)	177 (38)	30 (58)	153 (37)	24 (44)	136 (35)	17 (59)
Mechanical ventilation: N(%)	83 (9)	44 (7)	39 (13)	33 (6)	11 (13)	28 (6)	5 (10)	23 (6)	5 (9)	19 (5)	4 (14)
Percentage of hospitalisation time with at least one exit for procedures: median (IQR)	13 (0-22)	13 (0-22)	13 (0-20)	14 (0-22)	12 (3-19)	14 (0-24)	11 (0-17)	15 (0-25)	7 (0-14)	15 (0-25)	17 (0-25)
Ward-related variables (average): median (IQR)											
Patient age (years)	71 (69-72)	71 (69-72)	71 (70-72)	70 (69-72)	71 (70-72)	70 (69-72)	71 (70-72)	70 (69-72)	71 (69-72)	70 (69-72)	70 (70-71)
Infected patients(%)	72 (68-76)	71 (68-76)	72 (68-76)	71 (68-75)	72 (68-76)	71 (67-75)	72 (68-76)	71 (67-75)	71 (68-75)	71 (67-75)	73 (69-75)
Patients with immune-mediated disorders(%)	17 (13-23)	18 (13-24)	16 (12-23)	18 (13-24)	19 (12-24)	18 (13-24)	20 (13-24)	18 (13-24)	17 (13-24)	18 (12-24)	16 (13-22)
Patients with upper gastrointestinal tract disorders%)	10 (7-14)	10 (7-14)	10 (6-14)	10 (7-14)	10 (6-13)	10 (7-14)	9 (6-13)	11 (7-15)	10 (7-13)	11 (7-15)	8 (4-11)
Patients receiving oxygen without mechanical ventilation(%)	51 (40-55)	50 (39-55)	52 (41-55)	50 (39-55)	51 (40-57)	50 (39-55)	52 (44-56)	50 (39-55)	50 (38-54)	49 (39-55)	52 (41-57)
Mechanically ventilated patients (%)	12 (7-14)	11 (7-14)	12 (8-14)	11 (7-14)	11 (8-14)	11 (7-14)	12 (4-15)	11 (7-14)	11 (8-14)	11 (7-14)	12 (7-16)

Supplemental Table S6: annual mortality rates by categorical factors

Category	Value	N at baseline	Year post-discharge				
			1	2	3	4	5
All patients	NA	896	32%	14%	10%	12%	7%
Sex	F	376	28%	11%	9%	10%	7%
	M	520	36%	16%	11%	13%	7%
ADL/IADL dependence	YES	322	48%	24%	12%	11%	11%
	NO	574	24%	10%	9%	12%	6%
Psychiatric disorders	YES	79	32%	11%	6%	7%	10%
	NO	817	32%	14%	10%	12%	7%
Neurological disorders	YES	340	39%	21%	12%	12%	9%
	NO	556	28%	10%	9%	12%	6%
Endocrine/metabolic disorders	YES	347	35%	16%	13%	16%	9%
	NO	549	30%	13%	8%	9%	6%
Hypertension	YES	481	35%	15%	13%	13%	8%
	NO	415	29%	12%	7%	11%	6%
Infections on admission	YES	601	33%	13%	12%	14%	8%
	NO	295	31%	16%	6%	8%	6%
Cardiac disorders	YES	469	35%	19%	14%	17%	12%
	NO	427	29%	9%	6%	8%	3%
Cardiovascular disorders	YES	622	34%	16%	12%	14%	10%
	NO	274	28%	10%	6%	8%	1%
Pulmonary disorders	YES	435	37%	17%	12%	14%	9%
	NO	461	28%	11%	9%	10%	5%
Renal disorders	YES	294	39%	14%	14%	16%	7%
	NO	602	29%	14%	8%	10%	7%
Liver Disorders	YES	135	43%	14%	9%	15%	6%
	NO	761	30%	14%	10%	11%	7%
Immune-mediated disorders	YES	162	20%	6%	11%	6%	2%
	NO	734	35%	16%	10%	13%	9%
Cancer	YES	276	52%	19%	21%	13%	8%
	NO	620	24%	12%	7%	11%	7%
End-stage Cancer	YES	52	79%	27%	0%	13%	0%
	NO	844	30%	14%	10%	12%	7%
Vascular/haematological disorders	YES	307	31%	15%	9%	12%	4%
	NO	589	33%	13%	10%	11%	8%
Ophthalmological/ear-nose-throat disorders	YES	61	33%	5%	10%	11%	6%
	NO	835	32%	15%	10%	12%	7%
Upper gastrointestinal disorders	YES	72	44%	20%	9%	17%	21%
	NO	824	31%	13%	10%	11%	6%
Lower Gastrointestinal disorders	YES	80	33%	15%	17%	11%	6%
	NO	816	32%	14%	9%	12%	7%
Genital/urinary disorders	YES	98	32%	15%	18%	13%	5%
	NO	798	32%	14%	9%	12%	7%
Musculoskeletal/skin disorders	YES	185	29%	15%	7%	11%	6%
	NO	711	33%	14%	11%	12%	7%
Admission from nursing homes / ICU	YES	79	37%	10%	2%	14%	5%
	NO	817	32%	14%	11%	12%	7%
Immunodepression	YES	191	40%	8%	14%	12%	4%
	NO	705	30%	15%	9%	12%	8%
Need for surgery	YES	17	24%	15%	18%	22%	0%
	NO	879	33%	14%	10%	11%	7%
Oxygen supply without NIV	YES	401	36%	19%	14%	14%	11%
	NO	495	29%	10%	7%	11%	5%
NIV	YES	83	47%	25%	15%	18%	17%
	NO	813	31%	13%	10%	11%	6%
Vital parameters monitoring	YES	873	33%	14%	10%	12%	7%
	NO	23	4%	14%	16%	13%	0%
Intravenous drugs	YES	830	34%	15%	11%	12%	8%
	NO	66	17%	5%	4%	6%	0%
Vasoactive drugs	YES	10	50%	20%	0%	0%	25%
	NO	886	32%	14%	10%	12%	7%
Dyalysis	YES	13	54%	0%	0%	17%	0%
	NO	883	32%	14%	10%	12%	7%
Any oxygen support	YES	478	38%	20%	15%	14%	12%
	NO	418	26%	8%	6%	10%	3%
At least one nosocomial infection	YES	112	44%	13%	16%	13%	13%
	NO	781	30%	14%	9%	12%	6%

Supplemental Table S7: Deaths among frail§ and non-frail patients

	**3-months**		**6-months**		**12-months**		**24-months**		**36-months**		**48-months**		**60-months**	
	non-frail	frail	non-frail	frail	non-frail	frail	non-frail	frail	non-frail	frail	non-frail	frail	non-frail	frail
Deaths N(%)	47 (11)	98 (21)***	72 (17)	149 (32)***	95 (22)	195 (41)***	128 (30)	246 (52)***	144 (34)	282 (60)***	172 (40)	309 (66)***	185 (43)	325 (69)***

§ frailty index>0.25

Symbols

*:p<0.05; **:p<0.010, ***:p<0.001

Abbreviations

ADL – activities of daily living,

Supplemental Table S8: alternative model for associations with mortality by timeframe at multivariate logistic regression

Variable	Early mortality		Intermediate mortality		Late mortality	
	p	RR (95% CI)	p	RR (95% CI)	p	RR (95% CI)
Demographics						
Age	<0.001	1.04 (1.03-1.06)	<0.001	1.06 (1.04-1.07)	0.001	1.06 (1.02-1.1)
Sex	0.059	-	0.111	-	0.954	-
Nosocomial infections during hospitalisation	0.397	-	0.667	-	0.500	-
Causes of morbidity						
ADL/IADL dependence	0.002	1.92 (1.26-2.9)	0.838	-	0.896	-
Cancer	<0.001	5.20 (3.4-7.95)	<0.001	2.57 (1.61-4.1)	0.464	-
Immune-mediated disorders	0.07	-	0.677	-	0.258	-
Immunodepression	0.025	1.78 (1.08-2.94)	0.440	-	0.988	-
CIRS-SI score	<0.001	3.80 (2.04-7.08)	<0.001	4.03 (2.07-7.85)	0.207	-
Intensity of care						
NEMS score	0.002	1.07 (1.03-1.12)	0.629	-	0.042	1.1 (1-1.2)
Ward-related variables (average)						
Infected patients	0.484	-	0.448	-	0.14	-
Patients with immune-mediated disorders	0.383	-	0.982	-	0.456	-
Patients with upper gastrointestinal tract disorders	0.388	-	0.103	-	0.406	-
Patients with genito-urinary tract disorders	0.980	-	0.536	-	0.86	-
Patients with renal disorders	0.268	-	0.451	-	0.343	-
Patients with vascular/haematological disorders	0.433	-	0.853	-	0.288	-
Constant	0.001	0.00 (0.00-0.1)	<0.001	0.00 (0.00-0.07)	0.004	0.00 (0.00-0.02)

Abbreviations
ADL/IADL: general or instrumental activities of daily living. CIRS: cumulative illness rating scale. NEMS: nine equivalents of manpower score

Supplemental Figure S1 – Schematic representation of individual and Unit variables

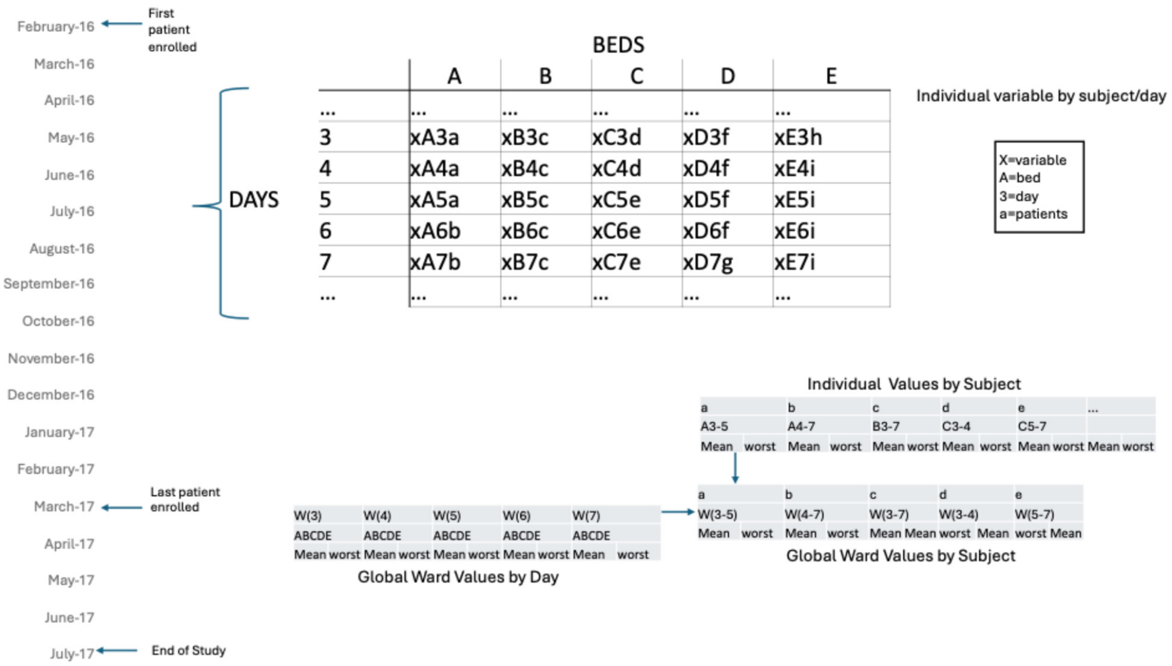

This figure depicts the methodology employed to record and derive individual- and Unit-level variables in the SIM cohort (Ramirez GA, J Personalized Med, 2024). On the left side of the figure, the timeframe in which the study was conducted is represented. In the central and right aspects of the figure, a representative variable of interest is labelled with "x", days by numbers, individual patients by lowercase letters and beds by capital letters. For each day any x variable, was collected per individual patients/occupied bed. Individual mean and worst value were calculated per each patient during the hospitalization. Ward means and worse values were calculated per each day integrating data from all occupied bed in a given day. Finally, for each subject during its hospitalization timeframe, the mean and worst variable values of the whole unit were also differentially calculated.

Supplemental Figure S2: heatmap of the strength of association among death and individual clinical and healthcare-related factors (univariate analysis)

	Early mortality	Intermediate mortality	Late mortality
Demographics and outcomes			
Age	<0.001	<0.001	<0.001
Sex	0.016	0.029	0.848
Nosocomial infections during hospitalisation	0.005	0.368	0.182
Causes of morbidity			
ADL/IADL dependence	<0.001	0.004	0.113
Cardiovascular disorders	0.049	0.001	<0.001
Hypertension	0.056	0.027	0.564
Cardiac disorders	0.045	<0.001	0.001
Lung disease	0.009	0.005	0.127
Kidney disease	0.002	0.022	0.831
Liver disease	0.004	0.649	0.9999
Cancer	<0.001	<0.001	0.649
End-stage cancer	<0.001	0.748	0.999
Immune-mediated disorders	<0.001	0.011	0.023
Endocrine-metabolic disorders	0.124	0.003	0.218
Neurologic disorders / dementia	0.001	0.008	0.215
Infectious disease	0.496	0.085	0.571
Upper GI disorders	0.022	0.232	0.019
Immunodepression	0.008	0.835	0.325
CIRS score			
CIRS total score	<0.001	<0.001	0.068
CIRS severity score	<0.001	<0.001	0.071
CIRS comorbidity score	<0.001	<0.001	0.038
Intensity of care			
NEMS score	<0.001	<0.001	<0.001
Any respiratory support	<0.001	<0.001	0.001
Oxygen without mechanical ventilation	0.02	<0.001	0.016
Mechanical ventilation	0.003	0.016	0.067
Percentage of hospitalisation time with at least one exit for procedures	0.114	0.001	0.876
Ward-related variables (average)			
Infected patients	0.037	0.586	0.115
Patients with immune-mediated disorders	0.022	0.832	0.665
Patients with upper gastrointestinal tract disorders	0.863	0.021	0.013
Patients with genito-urinary tract disorders	0.283	0.324	0.042
Patients with renal disorders	0.969	0.822	0.043
Patients with vascular/haematological disorders	0.533	0.962	0.011

This figure summarises the relative contribution of selected variables to early (year 1), intermediate (year 2-4) and late (year 5) post-discharge mortality. The reported values are the p-values of univariate chi-square (categorical variables) or Mann-Whitney U tests (quantitative variables) performed to compare the relative distribution of each category or quantitative dimension among patients dying or surviving at each timeframe of interest. More intense colours correspond to lower p-values. Non-significant values are depicted in grey font with white background.

Abbreviations
ADL/IADL: general or instrumental activities of daily living. CIRS: cumulative illness rating scale. NEMS: nine equivalents of manpower score
